# Forms of Joint Disease Met with in Medical Practice
1Paper read at a meeting of the Bath and Bristol Branch of the British Medical Association on April 27th, 1904, at Bath.


**Published:** 1904-06

**Authors:** Wm. Hale White

**Affiliations:** Physician and Lecturer on Medicine, Guy's Hospital, London


"*Scire est nescire, nisi id me*
*Scire alius sciret.*"

JUNE, 1904.

### FORMS OF JOINT DISEASE MET WITH IN MEDICAL PRACTICE.[1]

BY

WM. HALE WHITE, M.D. Lond., F.R.C.P.,
*Physician and Lecturer on Medicine, Guy's Hospital, London.*

RHEUMATIC fever is a definite disease having its own specific micro-organism and constant striking clinical characters, yet mistakes about it are not infrequent; and the error nearly always is, that some other disease is called rheumatic fever, and not that rheumatic fever is called some other disease. I believe the reason for this to be that rheumatic fever is so common and as a rule so readily distinguished that when, after easily and successfully diagnosing say forty-nine cases, the fiftieth difficult case presents itself we have become so accustomed to an easy diagnosis that we fall into error. This being so, I hope it will not be unprofitable if I give you an account of some mistakes I have known to be made.

[1] Paper read at a meeting of the Bath and Bristol Branch of the British Medical Association on April 27th, 1904, at Bath.

The first group illustrates the danger of diagnosing rheumatic fever when symptoms of this disease are present in a solitary joint. I believe the diagnosis of rheumatic fever should always be withheld so long as only one joint is implicated. Early in the disease a single joint may be affected, but while this is so leave the diagnosis in doubt. If the patient is suffering from this disease a second joint will be affected within twenty-four hours. I have twice known fracture into the knee-joint mistaken for rheumatic fever. I have seen the same error made in a case of osteo-myelitis of the head of the tibia, and this reminds me of a case which hardly illustrates the point under discussion, but is worthy of mention, for the patient had osteo-myelitis of the head of each tibia, with effusion into each knee-joint, but rheumatic fever was diagnosed. In a young girl acute necrosis of the head of the femur, with effusion of pus into the hip-joint, was regarded as rheumatic fever. In all these cases the error was recognised in time; each patient was handed over to a surgeon, and ultimately got well. But four years ago I saw a child who was suddenly taken ill with pain in the hip and fever. He was thought to be suffering from rheumatic fever, and was admitted into the hospital five days after the onset of the illness. He was then in the typhoid state, owing to septicæmia, and in spite of cutting down on the diseased hip immediately after admission to the hospital, he died. On several occasions I have known pleurisy or pneumonia to cause such pain in the shoulder on the same side as the pulmonary disease that the patient has been thought, as pyrexia was present, to be suffering from rheumatic fever. It is true that usually the pleurisy or pneumonia has been discovered, but the case has been wrongly regarded as rheumatic fever with rheumatic pleurisy instead of, as it ought to have been, pneumococcal pleurisy or pneumonia with reflex pain in the shoulder. On the other hand, I have once known pneumonia completely missed because attention had been so concentrated on the pain in the shoulder, which was thought to be rheumatic. In these cases the pain is not due to pneumococcal affection of the joint, for no effusion ultimately occurs. I have known many instances besides those quoted in which refusal to diagnose rheumatic fever when only one joint is affected has saved a mistake, but we will now pass on to other errors in connection with rheumatic fever.

It may be mistaken for almost any specific fever, because the patient who has fever often complains of pain in the limbs, and if these pains are referred to the joints an erroneous diagnosis may be made. Perhaps the mistake is commonest with influenza, but I have heard even of small-pox being mistaken for rheumatic fever; but in most cases the lapse of a few days sets the diagnosis right. This is often not so when pyæmia is mistaken for rheumatic fever. Such an error is by no means uncommon, and so important is it that it is worth while to mention a few examples. Some years ago a youth was transferred to my care from the surgical ward, where pain and effusion into several joints had led to a diagnosis of rheumatic fever. The discovery of some pneumonia, amongst other reasons, made us doubt this diagnosis. A rectal examination revealed a prostatic abscess, which was the source of the pyæmia. On another occasion a patient was sent in as a case of rheumatic fever, but a careful examination showed him to be suffering from pyæmia secondary to a sore on the heel; and in another instance rheumatic fever was incorrectly diagnosed in a patient suffering from pyæmia due to suppuration of both middle ears. In these cases the diagnosis of pyæmia made during life was unfortunately confirmed by a *post-mortem* examination; but in another instance a patient who was sent in from surgical out-patients as rheumatic fever recovered, after pus had been let out from the shoulder-joint, from the pyæmia from which he was really suffering, and which was due to a cut on the hand. The confusion between rheumatic fever and pyæmia is very serious; for if pyæmia is called rheumatic fever, failure to adopt early surgical treatment may cost the patient his life, and an almost certain hope of his recovery may be held out when he is within a few days of his death. When the pyæmia takes the form of malignant endocarditis the diagnosis may be difficult, as the following case will show:—

A boy, aged 11, was admitted under me on January 25th, 1895. He was one of a large family, several of whom had had rheumatic fever and chorea, and he had had both two years ago. On admission he had much pain in both knees; his temperature was 104.4°; he had systolic and presystolic mitral murmurs; he was thought to have rheumatic fever, but the diagnosis was regarded as doubtful as the skin was so dry. In less than forty-eight hours definite signs of meningitis enabled a diagnosis of malignant endocarditis, with pyæmic infection of the joints and meninges, to be made. He died six days after admission. Malignant endocarditis, suppurative meningitis, and pus in both knee-joints was found.

More than once I have known syphilis, which has caused effusion into the joints, to be mistaken for rheumatic fever. The following is an example of this error:—

A man, aged 26, was sent into the hospital under my care with a diagnosis of rheumatic fever, which he had also had twelve years before. The present illness began three weeks before admission with pain and swelling in the left elbow. A week later the left knee was affected in the same way, and soon after the left foot. On admission he had pyrexia, the above-mentioned joints were swollen, and he was sweating considerably; there was slight but distinct evidence of a secondary syphilitic rash; he had a sore throat, and had had a hard chancre ten weeks ago, and the arthritis was thought to be syphilitic. Forty-eight hours' treatment by salicylates made no impression on the disease, but he finally recovered with mercury.

Cerebro-spinal meningitis due to the diplococcus intracellularis may be mistaken for rheumatic fever. Pyrexia with pain and effusion into the joints are early symptoms in this form of meningitis. The rash may be thought to be a rheumatic erythema, and the pain caused by retraction of the head may be set down to rheumatism. I have known the mistake made.

Gonorrhœal rheumatism is frequently mistaken for other forms of chronic rheumatism; but if very severe it may be, and often is, thought to be rheumatic fever, for in a severe case of gonorrhœal rheumatism the temperature may be 103°. I saw this mistake made last month. The patient had pyrexia, but the facts that the joint trouble had persisted in a few joints for some days, that one knee was enormously swollen, that there was no blush over the joints, and the skin was dry made me suspicious. We found a slight gleet, and the case ran the course of gonorrhœal rheumatism. Sometimes help may be obtained from the fact that the effusion is in the sheaths of tendons as well as in the joints, for this is far more common in gonorrhœal rheumatism than in rheumatic fever. When gonorrhœal rheumatism occurs in women it is particularly likely to be overlooked.

It might at first be thought that typhoid fever could not be mistaken for rheumatic fever, but both are so common that it is not rare, if the febrile pains of typhoid are referred to the joints, for rheumatic fever to be suspected. Arthritis is so exceptionally a complication of typhoid fever that serious difficulty does not often occur; but it happened to me once to see a child, aged 6, who eight days before had been seized with sudden pain in the right hip and a temperature of 103°. The pyrexia persisted; the pain in a few days left the right hip and appeared in the left, and then the knees and ankles were painful and swollen. This led to a diagnosis of rheumatic fever; but when I saw the child the tongue, the spleen, and some typical rose spots were quite characteristic of typhoid fever, and that the disease was not rheumatic fever was proved by the fact that the left hip became completely disorganised.

Although some text-books apprehend difficulties of diagnosis between pneumonia and rheumatic fever, they do not, I think, often occur. Pneumococcal arthritis is a rare complication of pneumonia, but pneumonia is so common that we see the arthritis from time to time. A good instance is that of a child admitted with pneumonia. There was much diarrhœa, probably due to pneumococcal enteritis; a pneumococcal empyema formed, and was drained; both shoulder-joints swelled—pus containing a pure cultivation of pneumococci was found in each, and let out. The child got quite well.

Glanders has been mistaken for rheumatic fever, but fortunately it is so rare that the mistake is not common. The confusion between rheumatic fever and the acute joint affections in dysentery, Malta fever, and dengue does not concern us here, and acute rheumatoid arthritis will be best considered when we come to chronic rheumatism.

Acute gout is frequently thought to be rheumatic fever—an unpardonable mistake if deposits of urate of soda can be seen anywhere, but often when they are absent it is forgotten that gout may occur in poor people who are perfectly abstemious; indeed, I think that the worst cases of gout now seen are in those who are most careful in what they eat and drink, obeying their doctor punctiliously, for most of the cases that can be controlled by diet are so controlled. I need not take up the time of such a Society as this with the points of distinction. A day or two's observation will nearly always resolve any difficulties. That I am not alone in thinking the mistake common is shown by a recent paper by Futcher,[1] of Baltimore, who says: "I am quite sure that the apparent infrequency of gout in the United States is due to the failure of the profession to recognise the disease. The affection is usually mistaken for rheumatism."

I need not do more than mention the joint affections of scarlet fever or those that follow the injection of antitoxin, for the previous history always prevents mistakes.

It is so well known that when rheumatic fever attacks children the articular changes are usually slight, that although the lesser degrees of acute rheumatism are often overlooked in children, yet it is, I think, rare for a child to be said to be suffering from rheumatic fever when it is not. Mistake is most likely to occur in connection with scurvy-rickets, congenital syphilis, and the disease described by Still, in which effusion into joints is associated with enlargement of the lymphatic glands and spleen.

There is, I believe, with some of us a tendency to exaggerate the frequency with which rheumatic fever gives rise to chronic deformity of joints. Patients who have had this fever often suffer from very mild attacks, shown only by articular pain, with perhaps very trifling effusion and slight pyrexia; but serious deformity of joints is excessively rare, and, as far as my experience goes, is seen mostly in children. It is then recognised as being the result of acute rheumatism by the presence of other rheumatic affections, as chorea or endocarditis. The two following cases illustrate this:—

[1] *Practitioner*, 1903, lxxi. 6.

A boy, aged 12, was admitted under me. He had been in the hospital at the age of 5 with chorea; at the age of 7 with pains and slight swelling in several joints, endocarditis and pyrexia; at the age of 8 with erythema, fresh endocarditis, pain and swelling of several joints, relieved by salicylates. When admitted at the age of 12 there was much muscular atrophy, with fixation of all the joints of all four limbs in a position of flexion; the movements of his neck and jaw were impaired. It is true that some of his lymphatic glands were enlarged, but the chorea, erythema, and endocarditis made us regard the trouble as rheumatic. Although it was suggested that he was an example of the disease of children described by Still, in which many joints are swollen and the lymphatic glands and spleen are enlarged, against this view is the fact that there was chronic inflammation of the scalp, which would explain the enlargement of the glands of the neck; and they were the ones principally enlarged, and his spleen was not enlarged. He died at the age of 20, in his own home, from phthisis. My house-physician, Dr. H. J. Starling, made a *post-mortem* examination. The lungs were riddled with cavities, the pericardium was adherent, there were vegetations on the mitral valve.

The second case is that of a boy, aged 12½, now under my care. He has had chorea and pains in his joints. He now has some muscular atrophy and chronic enlargement, with effusion, of the wrists and one ankle. The affection of these joints has lasted at least two months.

We now pass on to consider the chronic affection of joints; and the first thing is, I think, to try and differentiate the diseases that are commonly grouped together as one disease under the names of arthritis deformans, osteo-arthritis, and rheumatoid arthritis. I have elsewhere[1] gone into this in detail. Here it will suffice to say that three diseases at least are grouped together under these terms. There is the disease usually called osteo-arthritis, often confined to the hip-joint, and commonly seen in elderly men. Secondly, there is the disease frequently met with in women in middle life, in whom the trouble commonly begins in the terminal joints of the fingers and the carpo-metacarpal joints of the thumb. The deformity is considerable, and is ultimately largely due to bony outgrowth. In a large number of cases the trouble gradually spreads until many joints are implicated, and the woman may become a helpless cripple; and although in one sense she

[1] *Guy's Hosp. Rep.*, 1902, lvii. 9.

is severely ill, yet the disease is very slow, the symptoms are not acute, and there is no pyrexia. Thirdly, we much less often meet with cases occurring for the most part in women whose age is usually near to 20, in whom the disease begins, or is at any rate soon most prominent, in the proximal phalangeal joints, and is quite early strikingly seen in the wrists. It is markedly symmetrical in the two hands; the swelling is considerable and extends beyond the joints, so that there is a well-marked fusiform swelling about the affected inter-phalangeal joints, and a general swelling about the wrist and other joints; sometimes a creaking may be felt, due apparently to thickening of the synovial membrane. In the course of a very few weeks most of the joints in the body are affected, so that the woman is soon bedridden, unable to move fingers, thumbs, wrists, elbows, shoulders, and the corresponding joints of the lower extremity. The joints of the spine are often affected, so that turning in bed is difficult and painful; the temporo-maxillary joint is frequently implicated, and mastication is consequently difficult.

Wherever the disposition of surrounding muscles makes it easy to investigate the joints swelling about them is obvious, and the patient usually complains of pain in them. Muscular wasting is so very rapid, that it is clearly an extreme degree of genuine arthritic atrophy not dependent on disuse; the wasting is especially seen in the hand and forearm, but in a bad case it is almost universal. The temperature is often raised daily, mostly in the evening, to a point usually between 100° and 102° F. The hands and feet are often covered with sweat; the patient is pale, and the pulse is increased in frequency out of proportion to and apart from pyrexia, although neither from the history nor from examination of the heart is there any evidence to connect the disease with rheumatic fever. Sometimes a number of brown spots, like large dark freckles, appear on the face, body, and limbs.

After two, three, or four weeks the temperature slowly regains its normal point; the rate of the pulse diminishes, but more slowly than the temperature; the colour partly returns, the sweating of the hands and feet lessens; the pain passes away, but much immobility and some swelling around the joints remain, and the patient is liable to fresh attacks, which exactly resemble the first and result in fresh swelling of and around the already fixed joints. After the acute stage has subsided the sufferer is by no means recovered, for the swelling of the synovial membrane and soft tissues around the joints leads to great immobility of them, and much of the muscular atrophy remains; the patient is hence often a chronic invalid. In this respect, as well as in others, the disease stands in striking contrast to nearly all cases of rheumatic fever.

Careful manual examination during life, even in cases which have lasted many years, fails to reveal any lipping or bony outgrowths in connection with the joints. The deformity appears to be entirely due to effusion into the joint and swelling of the tissues around. Radiographic examination entirely confirms this view.

This is an account of a severe attack, but these acute attacks frequently recur in the same patient; swelling then takes place in joints deformed from previous attacks. Some of the attacks may be less severe, the pyrexia only lasting a week or ten days. As a rule attacks diminish in severity as time goes on, and ultimately the patient may only suffer from the deformities due to previous attacks.

I have been fortunate in obtaining a *post-mortem* examination upon a patient affected with the disease; it is fully described in the paper in the *Guy's Hospital Reports.* Many joints were opened, and we were able to prove that the disease was one of the synovial membrane. There were no outgrowths of bone or cartilage. I show you a drawing from a microscopical section of a severely-affected phalangeal joint, and you will see that the synovial membrane is thickened by the formation of new fibrous tissue, which is chronically inflamed. You will see that the cartilage is only thinned and eroded in one place, just where it has been subject to the pressure of the fringe of thickened synovial membrane, and that just there and nowhere else there is a little inflammation of bone. It is clear that we have to do with a disease of the synovial membrane. Any affection of bone or cartilage is quite unimportant, and only secondary to pressure of thickened synovial membrane; and hence if any destruction of cartilage takes place it is not until years have elapsed, and then probably only occurs in one joint out of the many affected.

I have a number of photographs from three patients affected with the disease, and the condition of hands is very well shown, the spindle-like enlargement of the first inter-phalangeal joints being especially marked; but the affection of other joints and the arthritic muscular atrophy are also very evident. I also show you a drawing of the section of an inter-phalangeal joint, and the section itself is under the microscope. Further, I am passing round two temperature charts with the rate of pulse, which illustrate admirably the pyrexia and increased rapidity of the pulse. We will now pass on to consider the Röntgen ray appearances in these three cases. The thing that strikes us on examining these pictures is the unusually transparent appearance of the ends of the bones. In the hands it is best seen on either side of the metacarpo-phalangeal joints, but it is also quite evident at the ends of the phalanges. In the elbow too it was noticeable, especially in the olecranon, and other radiographs from these patients showed it in the ends of the bones forming the shoulders, ankles, and knees. I have looked at many radiographs of healthy joints, and also at those taken from patients suffering from chronic osteo-arthritis; and although the ends of the shafts are often more transparent than the middle, comparison with my two cases does suggest that possibly in future an exceptional transparency to the Röntgen rays of the ends of the bones will be regarded as peculiar to acute rheumatoid arthritis when this disease is contrasted with those having a very similar name. Three cases are too few for a generalisation, but this suggestion gains force from the fact that in looking through a number of radiographs of hands I came across one in which transparency of the ends of the bones was striking. The radiograph was labelled "Chronic osteo-arthritis," and I was unable to trace the case; but the radiograph showed no trace of bony outgrowth or lipping, which in itself was suggestive that the disease from which the patient was suffering was not chronic osteo-arthritis, but acute rheumatoid arthritis, and this view was supported by the fact that there was extreme flexion of the phalanges at the proximal inter-phalangeal joints. Now, as in acute rheumatoid arthritis, these joints are most strikingly affected and the muscular atrophy is extreme. The secondary flexion due to contracture is especially likely to occur at the proximal inter-phalangeal joints. Again, the suggestion here made that this exceptional transparency of bone may some day be found to be one of the distinctions of acute rheumatoid arthritis from other diseases having a similar name is indirectly supported by Walsh, who says: "The rheumatoid bones look more fragile; in point of fact they are atrophied, a condition which renders them more penetrable to the rays. Indeed, from their appearance it may be pretty safely inferred that they are deficient in bone salts." Later on he speaks of their great transparency. The radiograph he publishes to illustrate the point is taken from a lady, aged 31, who had had the disease since the age of 13, and nearly every joint in her body was affected. But it is very likely that this patient was really suffering from acute rheumatoid arthritis; for I have pointed out that this disease usually occurs in young women, that its resulting deformities continue for many years and may affect every joint in the body. Whether this transparency is greater in acute rheumatoid arthritis than in other diseases of joints, such as tubercle, osteomalacia, and cancer, in which Walsh says it may occur, is a point well worthy of further investigation. The transparency of bone is not transitory, for the patient who was in the hospital twice showed it on each occasion.

Want of time prevents my fully discussing the bacteriology of this disease and the possibility of its being a chronic infection. I hope to do this on another occasion, but I cannot refrain from mentioning one case which I have followed up. The patient was a young woman who presented the typical symptoms of acute rheumatoid arthritis. As there was much discharge of pus in connection with dead stumps of teeth, it was suggested that she was really suffering from an infective arthritis. All the dead stumps were taken out, and the mouth was made thoroughly healthy. She left the hospital about a year ago, and a fortnight ago I wrote to her. Her reply was that the joints were no better. Indeed, I think for many reasons that even if ultimately acute rheumatoid arthritis is shown to be due to a micro-organism, as it probably will be, that micro-organism will not be the streptococcus associated with ordinary suppuration, for not only does this case tell against such a view, but the blood, the spleen, the mesenteric glands, and the fluid from various joints of my fatal case were all examined very carefully in the Guy's Hospital bacteriological laboratory by cultures and innoculations and no ordinary streptococci or staphylococci could be found. The case reported by Gask in the last volume of the Pathological Society does not tell against this view, for there was no *post-mortem* examination: only fluid removed from the joint during life was examined, and this was harmless when injected into animals. I should like the opinion of members present whether acute rheumatoid arthritis is not a distinct disease; its characters are, it is acute with pyrexia and an abnormally rapid pulse, it relapses, there is anæmia. The sweating, which is extreme, is almost confined to the hands and feet; it occurs in young women, has none of the associations of rheumatic fever, is a synovial disease, any destruction of bone or cartilage is merely secondary to pressure of thickened synovial membrane, the tissues round the joints are thickened, the proximal inter-phalangeal joints are most characteristically and constantly affected with a spindle swelling. The temporo-maxillary joint is generally implicated, and even if cases have lasted many years there is no erosion of cartilage beyond what might be called accidental from pressure of thickened synovial membrane. Then too, there is the radiographic transparency. We now pass on to some mistakes often made in connection with chronic diseases of joints.

There is no disease about which we are so slovenly as gout. No malady can be certainly said to be gout until urate of soda can be seen. Very often a skilled observer can say from the clinical characters of an arthritis, *e.g.* of the great toe, that the probability of its being gout are so great that for all practical purposes the diagnosis is certain, but very often an arthritis which presents but few of the clinical characters of gout is called gouty. This should never be done unless uratic deposits can be seen in some part of the body. In many cases of chronic arthritis it is impossible to say whether the disease is rheumatoid arthritis or gout, but this is no justification for the phrase rheumatic gout. A disease may be chronic osteo-arthritis or gout, but it cannot be both, although in rare instances the two diseases may be present in the same patient.

Gonorrhœal rheumatism is probably, next to gout, the chronic disease of joints which gives rise to most mistakes. As already mentioned I have often seen it called rheumatic fever. Although the affection of the temporo-maxillary joint and spine are common in gonorrhœal rheumatism and almost unknown in rheumatic fever, yet these are frequently affected in acute rheumatoid arthritis. The affection of the sheaths of tendons and fasciæ are important aids to the diagnosis of gonorrhœal rheumatism, which is especially liable to be overlooked in the Mediterranean where chronic affection of joints in connection with Malta fever is often seen. Many books do not lay sufficient strength on the fact that gonorrhœal rheumatism is often accompanied by a very severe degree of arthritic muscular atrophy. I have actually twice known cases to be called progressive muscular atrophy when really they were the arthritic atrophy of severe gonorrhœal rheumatism.

Speaking of gonorrhœal rheumatism brings to mind the fact that flat foot is often overlooked; patients say they have rheumatism, the doctor acquiesces in this diagnosis of a case that is really one of flat foot. I have often seen this mistake made.

As far as my experience goes, an error which is not unfrequently committed is to confound sciatica and chronic osteo-arthritis of the hip. The mistake is serious, for in most cases it makes a considerable difference in treatment. I remember a man who had been seen by many doctors, and had been assured he had osteo-arthritis of the hip. As he was passing through London Mr. Edmund Owen and I saw him together, and were able to demonstrate to his doctor he had sciatica. On the other hand, a patient was sent into the hospital the other day as sciatica, but he really had osteo-arthritis of the hip. The most useful point of diagnosis is to search carefully for tenderness over the sciatic nerve. This can always be demonstrated in some part of the course of it, or its branches, if sciatica is present, in which disease the muscular atrophy follows the distribution of the nerve, but in hip-joint disease it is an arthritic atrophy of the muscles which move the hip.

Much interest has been excited of late by the question whether some cases of chronic articular rheumatism may not be instances of tubercular disease of joints. Usually speaking, tubercular disease of a joint is quite unlike rheumatism, and with regard to the question whether some cases usually looked upon as rheumatic and affecting many joints are tubercular, the proof is very difficult, for it has been suggested that the subjects of tubercle are more liable than others to chronic rheumatism, that even if the joint trouble is tubercular it is so chronic that by the time the patient dies the tubercle bacilli in the arthritic tissues are dead; and, lastly, it may be that in some of the rare cases about which there is doubt some of the joints may be tubercular and others rheumatic. The subject is ably discussed by Edsell and Lavenson in the *American Journal of Medical Sciences* for December, 1903. No certain conclusion is reached, but the suggestion—which seems probable—is made that some at least of Still's cases were examples of chronic polyarticular tubercular arthritis. The most interesting case of this kind that has come under my notice is that of a woman, aged 40, who had phthisis with tubercle bacilli in the sputum. Her elbows and ankles, shoulders and wrists were affected with a disease which would, if she had been otherwise healthy, have been certainly regarded as rheumatoid arthritis. Only the most careful *post-mortem* examination could have settled the point, and for this there was no opportunity. Another striking instance was that of a man who had fixation of the left hip. There was some rheumatic change in two phalangeal joints of the fingers and one of the toes. Some thought the condition was tubercular, while others were sure it was not; but a tubercular abscess ultimately formed in connection with the hip.

It is now gradually becoming recognised that some chronic cases which would certainly formerly have been regarded as polyarticular rheumatism, are really chronic infective arthritis, due to septic absorption from some chronic abscess. I would suggest that whenever we are examining a case of supposed chronic rheumatism we should thoroughly search the body for some chronic abscess, which is, I think, especially liable to be overlooked when in connection with the teeth. When the patient is an elderly woman, and therefore just the sort of person likely to have chronic articular rheumatism, the mistake is easy. For example, I saw the other day an old lady who had pyuria, and it was thought chronic articular rheumatism, but we found that the joints had only been affected since the pyuria began, and that those implicated were the shoulders, ankles, and the right knee. The shoulders were nearly fixed, and there was slight grating in the right; the ankles and knees were swollen and tender. There was no affection of the hands or feet as is so common in chronic articular rheumatism.

Hypertrophic pulmonary osteo-arthropathy is probably an instance of chronic infective absorption from the dilated bronchial tubes or chronic cavities in the lungs, and is not likely to be overlooked if the disease is borne in mind.

Some rare diseases of joints are particularly liable to be mistaken for chronic articular rheumatism, because the disease of which they are a part is very infrequent. This is perhaps hardly true of tabes, for that is a common affection, but it is a good thing to test the knee jerk and pupil reflex in many cases of chronic articular disease. I have known a joint scraped for supposed tubercle when really the patient was suffering from syringomyelia. This would not have occurred if the patient had been examined for syringomyelia. But the most remarkable case I have met with in which a nervous affection of joints gave rise to difficulty was that of a man, aged 43, who was sent into the hospital under me for supposed rheumatic fever, which he said he had had on a former occasion. He had an apical murmur and some pyrexia. The lower extremities were very tender, and he said this was why he could not move them. The left knee was swollen, but as the skin was perfectly dry I doubted the diagnosis. Two days after admission the right ankle became swollen and red. Two days after an acute trophic bedsore developed; later the bladder was affected, and other trophic sores developed. The patient ultimately recovered from a severe attack of acute myelitis, which was the cause of his arthritic trouble.

Two rare diseases about which I have known serious mistakes made are hæmophilia and Henoch's purpura. Cutting into the joint of a patient affected with the first disease will probably lead to his death, and in the second disease if the doctor omits to recognise the cause of the pains in the joints he fails to warn the friends of the appearance of other symptoms and of the great tediousness of the case.

